# The Use of WALANT Technique in the Osteosynthesis of Ankle Fractures: A Clinical Case Video

**DOI:** 10.1002/ccr3.70267

**Published:** 2025-02-20

**Authors:** Grigorios Kastanis, Constantinos Chaniotakis, Mikela‐Rafaella Siligardou, Ioannis Stavrakakis, Petros Kapsetakis

**Affiliations:** ^1^ Orthopaedic Department Venizeleio General Hospital Crete Greece

**Keywords:** ankle fracture, hemostatic mixture, lateral malleolus fracture, local anesthesia, WALANT

## Abstract

The Wide‐Awake Local Anesthesia No Tourniquet (WALANT) technique enables the creation of a suitable surgical field without the need for a tourniquet, while also allowing for the evaluation of the ankle under physiological forces (active ankle movement) after fixation. Data on the use of the WALANT technique in open reduction and internal fixation of ankle fractures are limited. This case video is the first to be presented in the literature.

## 1 Case Video

**FIGURE 1:**
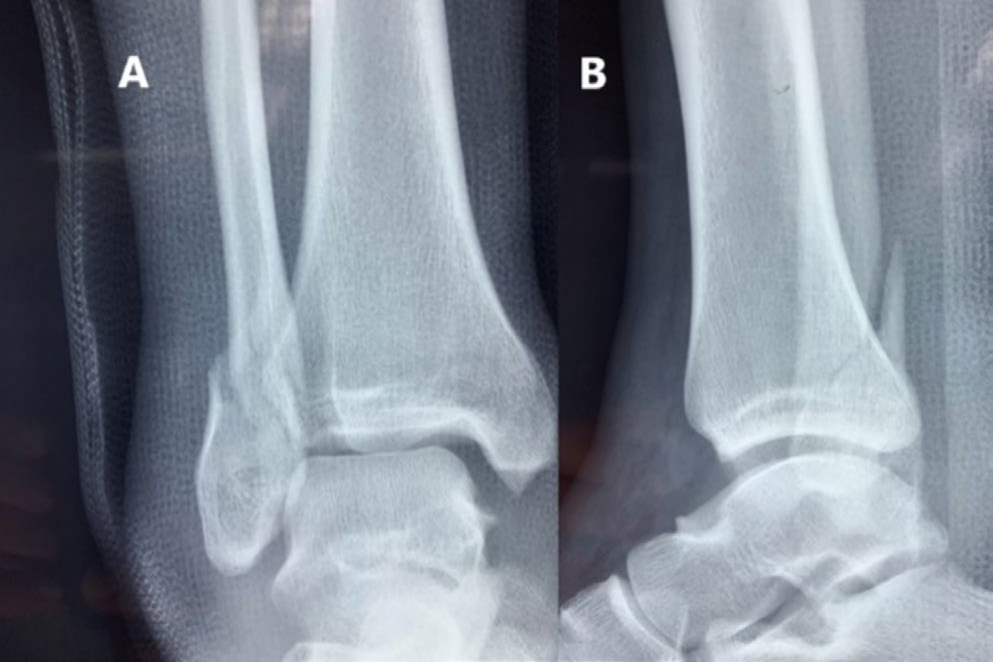
(A) Anteroposterior and (B) lateral preoperative x‐ray views of the right ankle, showing a lateral malleolar fracture and distal tibiofibular syndesmosis injury.

**FIGURE 2:**
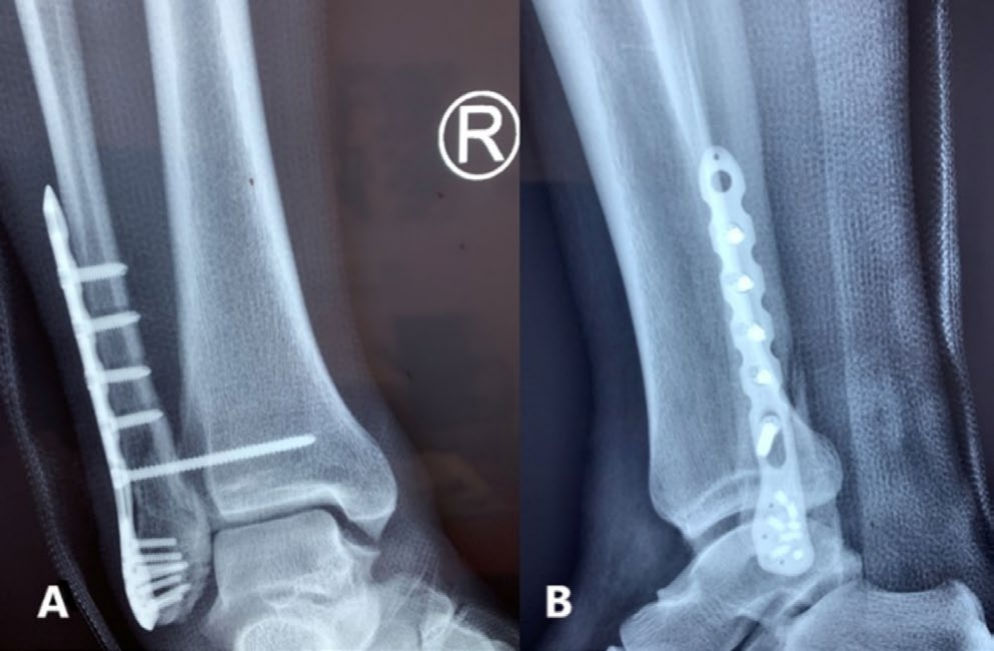
(A) Anteroposterior and (B) lateral postoperative x‐ray views of the ankle. open reduction and internal fixation of the lateral malleolar fracture with a plate and screws and placement of an ankle syndesmosis screw.

A 60‐year‐old male underwent open reduction and internal fixation of the lateral malleolus fracture with a plate and screws, along with the placement of an ankle syndesmosis screw, under local anesthesia using the Wide‐Awake Local Anesthesia No Tourniquet technique [1, 2] and without sedation (Figures 1 and 2). The components of the mixture (analgesic and hemostatic) used were 1% lidocaine with 1:100,000 adrenaline and 1 mL of 8.4% sodium bicarbonate. A total of 30 mL of this mixture was injected into the surgical field in successive stages of the procedure. The video focuses on the active intraoperative movements of the ankle after the completion of the osteosynthesis (Video 1).

**VIDEO 1:** The video shows intraoperative active movements of the ankle after the completion of the osteosynthesis. The patient was awake and followed the commands given to him intraoperatively. We were able to assess both active and passive movements of the ankle (ankle dorsiflexion, plantarflexion, inversion, eversion), as well as the stability of the osteosynthesis after its completion. Video content can be viewed at https://onlinelibrary.wiley.com/doi/10.1002/ccr3.70267

## Author Contributions

**Grigorios Kastanis:** conceptualization, data curation, formal analysis, investigation, methodology, supervision, validation, writing – original draft, writing – review and editing. **Constantinos Chaniotakis:** conceptualization, data curation, formal analysis, investigation, methodology, supervision, validation, writing – original draft, writing – review and editing. **Mikela‐Rafaella Siligardou:** data curation, investigation, methodology, validation, writing – review and editing. **Ioannis Stavrakakis:** data curation, investigation, methodology, validation, writing – review and editing. **Petros Kapsetakis:** data curation, investigation, methodology, validation, writing – review and editing.

## Ethics Statement

The authors have nothing to report.

## Consent

Written informed consent was obtained from the patient to publish this report in accordance with the journal's patient consent policy.

## Conflicts of Interest

The authors declare no conflicts of interest.

## Data Availability

All data generated or analyzed during this study are included in this article. Further inquiries can be directed to the corresponding author.
